# Professional Changes

**Published:** 1855-12

**Authors:** 


*Professional Changes.*—The chair of Obstetrics in the Medical College of Virginia, recently made vacant by the death of Prof. Bohannan, has been filled by the appointment of Dr. Conway, of Richmond. The chair of Chemistry in the College of Physicians and Surgeons, of New York, made vacant by the resignation of Prof. J. Torry, has been filled by the appointment of Prof. John Le Conte, of the University of Georgia.

Dr Alexander H. Stevens, venerable in years, and eminent for his professional attainments, has retired from the office of President of the same College; a station which he had honorably filled for many years.

Dr. T. Romeyn Beck, pre-eminent as a citizen, a teacher, and a man of science, died in Albany, New York, on the 18th of

Nevember 1855. Full of years and honors he has gone to the reward of his labors.

*Edinburg University:* Dr. Laycock, has recently been appointed to the chair of Practical Medicine in this ancient seat of medical learning. His competitors were Drs. Bennett, Craigie, Douglas, Gardner, McCormac, Munro, and Wood.

